# Be(e)coming pollinators: Beekeeping and perceptions of environmentalism in Massachusetts

**DOI:** 10.1371/journal.pone.0263281

**Published:** 2022-03-14

**Authors:** Sandra DiDonato, Brian J. Gareau

**Affiliations:** Sociology Department, Boston College, Chestnut Hill, MA, United States of America; Instituto Federal de Educacao Ciencia e Tecnologia Goiano - Campus Urutai, BRAZIL

## Abstract

In an era of mass extinction and biodiversity crisis, it is increasingly crucial to cultivate more just and inclusive multispecies futures. As mitigation and adaption efforts are formed in response to these crises, just transitions forward require intentional consideration of the hybrid entanglement of humans, human societies, and wider landscapes. We thus apply a critical hybridity framework to examine the entanglement of the pollinator crisis with the cultural and agricultural practice of hobbyist beekeeping. We draw on ethnographic engagements with Massachusetts beekeepers and find apiculture to be widely understood as a form of environmentalism—including as both a mitigation to and adaptation for the pollinator crisis. Illustrating how power-laden socioecological negotiations shape and reshape regional environments, we then discuss how this narrative relies on the capitalistic and instrumental logics characteristic of Capitalocene environmentalisms. These rationalities, which obscure the hybridity of landscapes, consequently increase the likelihood of problematic unintended consequences. Also present, however, is a deeper engagement with hybrid perspectives, with some beekeepers even offering pathways toward inclusive solutions. We conclude that if more just and biodiverse futures are to be realized, beekeeping communities must foster increasingly hybrid visions of apiculture as situated within socioecological and contested landscapes.

## Introduction

In an era marked by mass extinctions and biodiversity crises, it is increasingly crucial to intentionally cultivate more inclusive and just multispecies landscapes [1, 2]. Once complex and biodiverse ecologies throughout the world are facing mass homogenization due to ever-increasing threats from habitat loss, introduced species pressures, freshwater depletion, and terrestrial, aquatic, and atmospheric pollution—all of which also contribute to, and are exacerbated by, global climate change [3, 4]. As mitigation and adaption efforts are formed in response to these crises, we must intentionally consider the hybrid entanglement of humans, human societies, and wider landscapes; these problems must not be understood as environmental, but as socioecological [5]. As we respond to and coproduce our realities in a state of processual “becoming-with” the surrounding landscape [6, 7], therefore, we must develop “just transitions” forward with intention and care [1, 2, 8–11].

One specific area of concern, known as the pollinator crisis, refers to global findings that population and diversity decline among animal pollinators is being further exacerbated by decline and homogenization among angiosperms (flowering plants), and vice versa [12–14]. Eighty-seven percent of flowering plants, which represent eighty percent of all plants on Earth [15], require or benefit from animal pollination [13, 16, 17]. These contact zones [6] among pollinators and plants are also responsible for producing essential fruits, nuts, and vegetables for countless species—including humans. The relational landscapes that emerge from these spaces of contact are, therefore, essential sites for the continued existence of most terrestrial life—making the threat they face fully worthy of the term crisis [18–24].

Responsible for providing about ninety percent of all global pollination, many of the world’s 20,000 species of bees (Anthophila) face extreme threat from habitat loss, pollution, climate change, and more [16, 25, 26]. While all bee species face threats from numerous sides, most people in the United States are likely only aware of *Apis mellifera*—the most widely managed bee species in the world, commonly called the western honey bee [26]. Public awareness in the U.S. surrounding *Apis mellifera* can largely be traced to widespread media attention of mass colony loss among commercially managed hives in the mid-2000s, known as “Colony Collapse Disorder” (CCD) [19, 22–24]. CCD—though not reported within the last decade and now understood not as a disorder, but as the result of an amalgam of causes including poor nutrition, insecticide exposure, multiple diseases, and more [24]—nonetheless remains a strong motivator for why people become beekeepers [22, 24, 27]. Scholars have even found that a “new wave of beekeepers” participating in a global “urban beekeeping boom” has increased in recent years in part due to this desire to “save the bees” from CCD [28–31]. Instead of commercial beekeepers, who depend on profit gained from selling hive products or renting colonies for pollination [23, 32–35], this surge has occurred among hobbyists, beekeepers who do not sell hive products or pollination services, and sideliners, hobbyists who sell products or pollination services but may or may not make a profit [19, 22, 23, 36].

Scholars explain that continued concerns for CCD and honey bees in general have led to increased awareness for native bee concerns; the honey bee is considered a “canary in the coal mine” [14, 18, 37, 38], “mascot” [29], and “flagship species” [25, 39, 40] of wider bee and pollinator declines [14, 18, 25, 29, 37, 38]. In light of these claims, however, others argue that continued focus on *Apis mellifera* often distracts attention away from the specific needs of lesser known, yet vital, bee species [18, 24, 41]. Many hobbyists and sideliners, for example, believe that becoming a beekeeper not only helps honey bees, but also benefits the wider environment [29, 30, 34, 42]. Much of this beekeeping scholarship is explicitly framed with the concept of the Anthropocene [12, 28, 43, 44], which posits that the human species has so thoroughly defined the planet that we have now entered a “post-wild” world where humankind must carefully manage, steward [45], and garden the earth [46–49]. Beekeeping is thus widely framed as “an ecologically inspired urban lifestyle phenomenon” [29] and a “virtuous hobby…where humans work to save a vulnerable species and in the process…the planet” [30].

Critical scholars have deeply questioned the instrumental and objectifying rationalities at the root of this Anthropocene narrative, explaining that by framing dynamic landscapes as “conceptual and material resource for human world-making” [3], these rationalities are one and the same with those foundational to capitalist ideology [7, 10, 47, 50, 51]. With arguments that planetary degradations are also pointedly not caused by a generalized “humanity,” but rather by the application of these very modes of thought as they manifest in the current world ecology of capitalism, many critical hybridity scholars thus conclude that we are truly living in the era of the Capitalocene [10, 50, 52, 53].

When Capitalocene environmentalisms [52] are applied in conservation efforts, complex socioecological problems are thus addressed with the same rationality that created them in the first place [5, 54, 55]. O’Connor (1988) termed this process the “second contradiction” of capitalism, which he explains leads to an increased risk for unintended consequences and negative externalities, including social discord and environmental catastrophe [52, 56–58]. The entanglement of hobbyist beekeeping and the current biodiversity crisis provides a case to examine how these theoretical arguments play out on the ground. While beekeeping to “help the environment” remains a powerful narrative, entomologists and ecologists since the 1970s have provided evidence that beekeeping can have negative consequences for local wild bees and ecosystem biodiversity more generally—especially when colonies are located outside Africa, the Middle East, and Europe, where *Apis mellifera* is indigenous [37, 41, 59–68]. Sociological inquiry, however, has only begun to address these tensions between beekeeping and biodiversity conservation [69], and critical hybridity analyses of this topic are rarer still [25, Cf. 27, 28].

Following the tenants of critical hybridity to cultivate more inclusive just transitions [5] and “just sustainabilities” [8, 9, 70, 71] as we move forward with the damages of the Capitalocene [2], we thus fill this gap by looking beyond the hive to provide a critical hybridity analysis of beekeeping and biodiversity conservation. Using ethnographic methods, including participant observation, stakeholder interviews, and textual data gathered from the beekeeping community in the Commonwealth of Massachusetts, we investigate how beekeepers understand apiculture in the wider landscape. What dominant narratives are driving how beekeepers engage with and adapt to the wider landscape? Could hybrid and relational approaches to beekeeping provide pathways toward more just socioecological futures?

Our data are necessarily specific to Massachusetts communities in unique geographic, historical, and socioecological contexts—in addition to being specific to each participant’s unique and relational positionality in gender, racial/ethnic, economic, and other matrices of power. Our findings illuminate, however, broader socioecological processes regarding how capitalist modes of thought are already shaping the ways groups of people are understanding and responding to contemporary socioecological crises. In the Capitalocene, it is critical to be mindful and intentional about how our mitigating and adapting behaviors unfold in specific contexts. Our analysis thus emphasizes the importance of investigating contested contact zones where logics and ideologies impact the shape of material ecosystems at the local and regional scale. We conclude that if more just and biodiverse futures are to be realized, beekeeping communities must foster hybrid visions to see beyond the confines of Capitalocene logics toward understandings of apiculture as situated within hybrid, socioecological, and contested landscapes.

## Theoretical approach: Critical hybridity and the problem of instrumental rationality

Critical scholars have illustrated how the epistemological and ontological assumptions behind Anthropocene environmentalisms objectify and obscure the contested processes through which the “web of life” emerges [72]. These scholars further explain how these modes of thought are one and the same with those which provide the foundation of capitalist—or with regard to instrumentalism especially, neoliberal capitalist—ideology [5, 47, 51, 73–75]; “if a fixed Nature is required for authoritarian modes of conservation…a fluid, individualistic, and fungible nature is necessary for neoliberalism” [47]. The instrumental logics applied here are thus, “committed to fixing the parts and not the whole” [53], allowing for the framing of hybrid communities of organisms as separate entities of natural capital [76] and instruments of ecosystem services [see 77 for review].

Critical hybridity scholars, however, explain that far from generalizable services and natural processes, life actually emerges out of mutually inclusive, relational webs that “cannot be planned…cannot be controlled” [51]. Interpreting Haraway (2008) [6], White et. al (2016) explain, “relations, processes and interactions precede material entities and categories like ‘human’, ‘animal,’ ‘technical’…categories and materializations are produced by webs of interaction” [5].

Drawing from and expanding upon the pioneering work of Latour [78], Callon [79], Law [80], Haraway [81], and others [Cf. 82, 83], our approach to critical hybridity joins other critical scholarship that recognizes a “false antithesis” between hybrid approaches influenced by Actor-Network Theory and the radical approaches of Neil Smith [84], James O’Connor [56, 85, 86], and World-Systems Theory [53]. Here, critical hybridity acknowledges the agency of the multispecies landscape, weather patterns [Cf. 87], objects and tools of inquiry that make up actor-networks, while also remaining attentive to uneven levels of power and the socioecological structures of the Capitalocene. White et al. (2016) thus explain,

“…acknowledging that a relatively stabilized network of capitalist relations exist, that these capitalist networks are marked by inherent and patterned uneven development and crisis tendencies, that they presently have organized themselves in particularly rapacious neoliberal forms, and that none of this can be wished away or dissolved via studying actor networks or assemblages detached from political economic analysis” [5].

To best understand hybrid socioecological landscapes and develop just transitions forward, therefore, we must critically examine the relations at these highly contested and power-laden “contact zones” in which organisms “become-with” the surrounding landscape [2, 3, 5, 6, 81, 88–90].

With this relational ontology at the center, scholars have further developed concepts like “agentic assemblage” and “distributed agency” in order to theorize and discuss how agency and power move across hybrid landscapes [91]. As agency is distributed throughout the landscape, “the effects generated by an assemblage…emergent properties…distinct from the sum of the vital force of each materiality considered alone” [91]. Power, similar to agency, is also understood as having a “liquid” nature which “coagulates” in socioecological spaces across hybrid landscapes of humans, human societies and institutions, and multispecies, microscopic, and abiotic communities [5, 6, 56, 72, 85].

Due to the ubiquity of instrumental rationality, however, these considerations are often not taken into account. Complex socioecological problems, including historical approaches to pollination concerns thus far, are instead often addressed with the same capitalist rationality by which they were created [5, 54, 55].

## Background: Apicultures in the Capitalocene

In the late 19^th^ century, after decades of widespread urban development and the increasing industrialization of agricultural practices, U.S. farmers began to notice widespread bee declines [19, 22–24]. In response, farmers began to introduce honey bees to their fields during crop bloom [22–24]. By 1929, the *New York Times* reported bee colony rental as a practice that had been active for “many years” [92]. Commercial honey bee pollination was later solidified as a widespread norm in the post-WWII era when commercial agriculture became increasingly dominated by high mechanization, monocultures, and largely unregulated agrochemicals [23, 93]. Some even argue, “it is only with the growing intensification of a highly industrialized and chemically dependent form of U.S. agriculture during the mid-twentieth century that growers began to see the virtue of honeybees as crop pollinators” [23]. Instead of addressing the root causes of pollination concerns, therefore, honey bees were widely introduced as an “instrument” of pollination—a powerful application of instrumental rationality that continues through today.

Today, millions of commercially managed bees are now transported across the United States annually—from almond orchards in California, to orange groves in Florida, to cranberry bogs in Massachusetts [28, 94]—in order to “fix” this pollination problem. This response in many ways supports the Capitalocene narrative, where solutions to environmental problems must fit the economic and cultural logic of capitalism [50, 57, 72]. In this case, pollination is addressed through the purchase of a commodified honey bee irrespective of or unconcerned about the potential negative externalities

The unintended consequences of commercial pollination, however, could not remain ignored. There has been great attention in recent decades toward the stresses these migrating honey bees face, including agrochemical poisoning, increased disease transmission, and many issues that result from tightly scheduled cross-country truck migration [19, 22, 95, 96]. Increased awareness of these stresses faced by migratory honey bees has led to a growing body of scholarship focused on the controversies among beekeepers, researchers, and the agrochemical industry [19, 22, 43, 97]. This scholarship includes a growing body of multispecies scholarship [CF. 4] on beekeeping, in which scholars apply “multispecies” frameworks [28, 30, 31], “more-than-human” methods [43, 97], and multispecies “whole of community” (WOC) approaches [12, 44]. Social scientists have also written on a variety of related topics including bee protection policies [34], disputes over access to forage resources [98], “simian-apian” assemblages [43], and even critical assessments focused on apiculture under industrial capitalism [28, 32, 95, 99]. It is now widely argued that increasing, or even sustaining, demand for large-scale commercial pollination is not only extremely harmful to honey bee health, but also unmanageable and precarious for farmers, beekeepers, others in the honey bee supply chain, consumers of industrially farmed food, and the wider hybrid landscape [19, 22, 23, 32, 43, 97, 99, 100].

While many often focus on these large-scale commercial pollination operations, hobbyist beekeepers and bees are also deeply enmeshed in commercial beekeeping processes. Hobbyist and commercial beekeepers alike, for example, often purchase bees from commercial rearing facilities which breed from bees that originated in the commercial pollination system. Further, although some beekeepers still hope to “save the bees” by beekeeping, honey bees are not at risk of extinction, and are actually growing in colony numbers largely due to this capitalist production process [18]. Regardless of low extinction risk, however, *Apis mellifera* still faces persistent stressors including agrochemical poisoning, like neonicotinoid exposure since the late 1990s [101], and the *Varroa destructor* (*Varroa*) mite since its introduction to the U.S. in the late 1980s [93].

### Hobbyist beekeeping and biodiversity conservation

Desires to help “save the [honey] bees” from various threats have also become deeply entangled with narratives regarding the pollinator crisis and native bee conservation. Even though honey bees are seen as the “canary in the coal mine” [14, 18, 37, 38], for example, most people in America remain largely unaware of species beyond *Apis mellifera* [18, 26, 102]. Even federal attention through the 2016 EPA “Pollinator Partnership Action Plan” (PPAP) has been highly honey bee centric [103]. This report lists three goals: (1) helping honey bees, (2) supporting monarch butterflies (*Danaus plexippus*) for their “iconic migration,” and (3) increasing pollinator habitat average. Even this third goal, which may seem closest to biodiversity conservation more broadly, is also narrowly focused on perceived services provided by honey bees and monarchs—habitat is to be increased with “natural forage to support over-summering honey bees in the upper Midwest, and pollinator habitat along the prairie plains States to support the migration of the Eastern population of the monarch butterfly” [104].

Scholars have highlighted that an over-emphasis on honey bees may distract attention from native bee needs [41, 102]; in contrast to honey bee colonies, for example, ninety percent of bees are considered “solitary” species that face many of the same threats as honey bees in addition to nuanced pressures on specific nesting, reproductive, and dietary needs [18, 26, 104]. Further, however, there is also evidence that honey bee colonies can also disrupt the complex relations from which local landscapes emerge. This concern can be broadly grouped into three interconnected tensions that may arise when managed honey bee colonies are introduced into wild ecosystems: (1) possible competition with native bees for nectar and pollen (forage) [105–114]; (2) increased likelihood and severity of pathogen transmission from managed honeybees to native bee communities [114–119]; and, (3) changes in composition of wild plant communities by both providing (honey bee) and deterring (native bee) pollination [68, 112, 120, 121].

With multiple plants indigenous to America and best pollinated by increasingly threatened species of native bumble and squash bees—including those in the genus *Vaccinium* (including blueberries and cranberries) and the genus *Cucurbita* (including squash and pumpkins) respectively [120, 122, 123]—these tensions are also being linked to fruit set decline among these crops in areas near honey bee hives [124]. This is partly because *Vaccinium* and other wild plant communities require pollination by vibration (known as sonication), a technique which is performed not by honey bees, but by native bees including bumble and sweat bees [125]. These tensions are also deeply entangled with one another—if honey bees successfully pollinate invasive plant species, for example, this can contribute to the suppressing of native plant populations and exacerbate the impact of competition or diseases on native bees in some cases [120, 126].

These concerns thus illustrate the relational entanglements between “two of the most problematic arenas of human-animal relations today in terms of environmental impacts: (1) industrial animal agriculture and (2) the decline of wild animal species (‘defaunation’)” [10]. These two arenas are deeply interconnected in historically specific ways. For example, given that honey bees not only live in highly social colonies, but are also commercially reared in large numbers [116, 117, 127], they are more likely to both outcompete and have a higher prevalence of pathogens than wild bee populations [128]. Concerns surrounding the influence of honey bees on local plant communities further illustrate these complexities; honey bees were first brought to North America through the “Columbian Exchange” [5, 129], or the process through which large-scale European colonization moved countless species of plants, animals, fungi, and microorganisms around the world. Many of the species brought to America—including honey bees and many plants—were indigenous to the general region surrounding Europe, the Middle East, and Asia [126, 130–132]. When honey bees and introduced plants are from similar geographic regions, these plant species are more likely to be effectivity pollinated by honey bees than by the native bees of new landscapes [68, 133, 134].

Hobbyist beekeeping is now considered a “gordian knot” given the extreme complexity of measuring ecological concerns against the positive benefits of participating in a beekeeping community [135]. After the highly acclaimed academic journal *Science* published the article “Conserving honey bees does not help wildlife” [37], these concerns for beekeeping and biodiversity conservation spread beyond the academe [e.g., 136, 137]. The dismissal or acceptance of these findings among beekeepers thus illustrates how powerful narratives and taken-for-granted epistemologies drive some landscapes to become privileged over others [6, 138].

### Beekeeping and the Massachusetts landscape

Massachusetts has a rich history of hobbyist beekeeping. The Massachusetts colony was the second to receive honey bees (1630–1633) [139] and the first to run a town apiary (Danvers, MA, 1640) [93]. Later, Massachusetts was the first state to commercially rear queen bees (1861) [139] and to start a county-level beekeepers’ association (Worcester County Beekeepers’ Association, in 1900) [140]. Massachusetts was also home to Reverend Lorenzo Langstroth of the Second Congregational Church of Greenfield, MA—who invented what remains the most widely used beekeeping configuration in the world, the removable frame hive or “Langstroth hive,” in 1852 [141].

The contemporary beekeeping culture in Massachusetts is no less vibrant. The town of Greenfield celebrates an annual Langstroth Bee Fest and the Topsfield Fair in Topsfield, MA continues to boast the largest honey show in America. Massachusetts beekeepers are also supported by a plethora of social institutions and federal, state, and local government programs. These include: the Massachusetts Department of Agricultural Resources (MDAR) Apiary Program; the state-wide Massachusetts Beekeepers’ Association (MassBee); eleven county-level beekeepers’ associations; extension honey bee researchers at the Center for Agriculture, Food, and the Environment at the University of Massachusetts (UMASS); nonprofit organizations (i.e., the Essex Agricultural Society, which runs the Topsfield Fair, and the Second Congregational Church of Greenfield, which runs the Langstroth Bee Fest in collaboration with the local beekeepers’ association); the Audubon Society (which operates apiaries for agricultural pollination, honey production, and educational programs); and for-profit organizations including beekeeping supply companies and full-service beekeeping providers (i.e., The Best Bees Company, henceforth Best Bees, founded in Boston). Among organizations in this network, beekeeping associations may be particularly important, as power often “coagulates” at social institutions as they facilitate communal procedures and norms [5].

In Massachusetts today, over 40,000 colonies are managed by approximately 4,000–4,500 beekeepers, nearly all of which are hobbyists and sideliners [36]. A speaker from MDAR at the 2020 Pollinators in Our Land Conference explained: *I have yet to find a single area in the state with a five-mile radius…without honey bees*…*People love honey bees*. *They love beekeeping*. Most beekeepers purchase bees from commercial rearing operations—including artificially inseminated queens, packages (bee colonies without a queen), and small colonies with a queen already introduced (nucleus colonies or *nucs*). Most keep their bees in Langstroth hives, in which mature colonies can be expected to have about 60,000–80,000 bee individuals [142]. A speaker at the 2019 MassBee Spring Meeting further reported an estimate of 1,000–1,500 new beekeepers in the state each year, though, the speaker elaborated: *a lot of [new beekeepers] will become ‘bee havers’ with good intentions and give up the hobby…with voluntary registration*, *there’s no way of knowing [the number of beekeepers for certain]*.

Unlike large scale commercial honey bee colonies, honey bees in Massachusetts are often kept at the same apiary location year-round, always present in the local environment and active outside the hive with dry conditions and air temperatures of at least 13°C/55°F [143]. In addition to colonies that survive the winter, many hobbyists also purchase new or replacement bees annually. Unlike native wild bees then, honey bees may always maintain a presence in Massachusetts as long as they are reared or available for sale. The distributed agency at play in the assemblage of hobbyist beekeeping must, therefore, be understood as a powerful and contested space in the shaping of Massachusetts landscapes.

Bees in Massachusetts, however, do not only include *Apis mellifera;* there are over 350 bee species indigenous to Massachusetts [123, 144]. Most of these bees are solitary species with short life spans that are specifically adapted to certain local ecological processes and conditions. Massachusetts is home, for example, to many “specialist” bees which have evolved closely with specific local plants and rely on them for survival [16, 120].

These endemic bee/plant contact zones, along with the rest of the hybrid Massachusetts landscape, are under extreme threat from habitat loss, pollution, pesticide use, and climate change—with air temperatures increasing in the Northeastern United States faster than the global trend of about 1°C [145]. As the third most densely populated state, bee habitat in Massachusetts remains particularly threatened by the state’s urbanization patterns, which have increased over the last century and accelerated in the last few decades [146–148]. Finally, pathogen transmission among wild bees in North America is seen as increasingly alarming for conservationists; for example, the microsporidian *Nosema bombi* was found to be heavily present among the communities of four North American bumble bee species that have declined by over 90% in the past two decades alone [104, 149].

Even with greater awareness of the specific needs of native ecosystems, and the tensions between beekeeping and biodiversity, however, the most prominent step the Commonwealth has taken for bee or insect conservation can also be characterized as highly honey bee and beekeeping centric. The 2016 “Massachusetts Pollinator Protection Plan” [36], for example, was prepared by the Apiary Program Working Group of the MDAR Division of Crop and Pest Services—not the conservation-driven Massachusetts Division of Fisheries and Wildlife (MassWildlife). The report is also focused largely on honey bees, which is to be expected given that the authors explicitly claim the report takes direction from previously discussed honey bee centric PPAP [104].

## Materials and methods

With a focus on the relational becoming-with of hybrid landscapes, therefore, we seek to critically engage with the rationalities and logics surrounding apiculture in the Massachusetts landscape—those which lead to environmentalisms rooted in Capitalocene logic, and thus result in problematic unintended consequences, and those which point towards socioecological, hybrid-informed just futures. To do this, we draw on data collected over eighteen months of fieldwork (October 2018 to March 2020), with a focus on observation of eleven bee-related events and twenty-two in-depth interviews with beekeepers (all participants are given pseudonyms; interview schedule in S1 Text). This study was approved by the Boston College Institutional Review Board (IRB) (#19.165.01e).

Events ranged from one-hour beekeeping school (*bee school*) classes to seven-hour beekeepers’ association meetings (S1 Table). Most events were run by MassBee and advertised in MassBee email communications, Facebook posts, and newsletter, *The Massachusetts Bee*. Events not directly run by the hobbyist community included an office visit to Best Bees; attendance at two Massachusetts film festival showings of *The Pollinators*, a documentary focused on migratory honey bee content and directed by Northeastern-based hobbyist beekeeper Peter Nelson; and attendance of the Pollinators in Our Land conference administered by The Center for Agriculture, Food, and the Environment extension office at University of Massachusetts (UMASS) Amherst.

Interviewees were recruited through beekeepers’ association Facebook pages and in-person events via “convenience sampling” [150]. Interviews lasted between one and two hours and were conducted using an in-depth “semi-structured” format, which allows for researchers to “stumble upon and further explore complex phenomena that may otherwise be hidden or unseen” [150]. We did not initiate discussions regarding specific conservation concerns and honey bees in the landscape, but instead, inquired about broader topics in order to provide beekeepers with some autonomy over the conversation. Interview questions focused on four themes: (1) practices, (2) social conditions, (3) awareness and opinions on beekeeping in the surrounding landscape, and (4) climate change. Demographic and value-based questions, such as gender, income, and political ideology, were asked at the end of the interview.

Before analysis, we employed NVivo transcription on interview recordings and subsequently imported transcripts, field notes, print materials, and a strategic sample of the ten issues of *The Massachusetts Bee* published during the 17-month fieldwork period, into NVivo 12 for analysis. Following the tenets of a critical hybridity approach, analyses of these data were performed alongside careful attention to natural science literatures regarding beekeeping and biodiversity conservation [5]. Data were first coded for major themes and subsequently coded using an iterative research process with particular attention to how participants engaged with beekeeping and the surrounding landscape [150].

All beekeeper interviewees were located in eastern Massachusetts during the study period with two exceptions—two married beekeepers from Rhode Island who are nonetheless active in the eastern Massachusetts beekeeping community (pseudonyms: Julie, a 65-year-old sideliner, and her husband Archie, a 68-year-old *assistant beekeeper*) and one beekeeper located west near Springfield, Massachusetts (pseudonym: Sabrina, a 62-year-old hobbyist and beekeeper at a nonprofit) (Table 1).

**Table 1:** Beekeeper demographics, operations, and social involvement at time of study.

Pseudonym	Beekeeper Label	Age	Gender Identity	Years^1^	Highest Education	Household Income	Political Ideology	Local area	Social involvement in association-centered beekeeping community
Ambrose	Sideliner	41	Male	9	Bachelor’s degree	$100-150K	*Undecided*	Spacious suburb	Highly active, holds leadership position
Bill	Hobbyist and employed at a nonprofit	71	Male	12	Bachelor’s degree	$100-150K	Liberal	Thickly settled suburb	Highly active, holds leadership position, advocate for “natural beekeeping"
Blake	Beekeeper at Best Bees	24	*Female and questioning*	4	Bachelor’s degree	$30-40K	Liberal	Urban	No membership with beekeeping associations
Brooke	Hobbyist	54	Female	2	Bachelor’s degree	Prefer not to answer	Prefer not to answer	Thickly settled suburb	Highly active, advocate for rehabilitation beekeeping programs
Elliot^2^	Hobbyist	20	Male	2	Associate degree	Prefer not to answer	Moderate- liberal	Suburb	Highly active, holds leadership position
Emmett	Hobbyist	36	Male	1	Bachelor’s degree	$100-150K	Libertarian	Spacious suburb	Active only on association Facebook page
Harvey	Sideliner	60	Male	N/A^3^	Master’s degree	$150K or more	*Socially liberal and fiscally conservative*	Rural	Active, attends meetings
Helene	Hobbyist	71	Female	16	Master’s degree	Prefer not to answer	Liberal	Suburb	Active, attends meetings
Hyde	Hobbyist and employed	25	Male	5	Bachelor’s degree	Less than $30K	Liberal	Spacious suburb	Active through employment
Isabella	Sideliner	42	Female	2	Associate degree	$150K or more	Conservative	Spacious suburb	Active, attends meetings
Jackie	Hobbyist	60	Female	N/A	Bachelor’s degree	$100-150K	Liberal	Suburb	Active, attends meetings
Jasper	Sideliner	60	Male	14	Bachelor’s degree	$100-150K	Moderate	Suburb	Active, attends meetings
Julie & Archie	Sideliner & *assistant beekeeper*	65 & 68	Female & Male	7	Master’s degrees	Prefer not to answer	Liberal	Spacious suburb	Very active, attended advanced beekeeping class
Kitty	Sideliner	63	Female	N/A	Bachelor’s degree	$150K or more	Moderate-liberal	Rural	Active, attends meetings
Leo	Sideliner	74	Male	6	Master’s degree	$75-100K	Conservative	Rural	Very active, membership to multiple associations, attends meetings, attended advanced beekeeping class
Liam	Sideliner	77	Male	13	Bachelor’s degree	$50-75K	Moderate- conservative	Suburb	Active, attends meetings
Marigold	Beekeeper at Best Bees	26	Female	2	Bachelor’s degree	$30-40K	Liberal	Urban	No membership with beekeeping associations
Nick	Hobbyist	75	Male	N/A	Some college, no degree	$100-150K	Moderate	Spacious suburb	Highly active, to attend advanced beekeeping class
Sabrina	Hobbyist and employed at a nonprofit	62	Female	3	N/A	$50-75K	Moderate-liberal	Suburb	Highly active
Sofia	Hobbyist	69	Female	8	Master’s degree	$100-150K	Prefer not to answer	Urban	Highly active, membership to over four beekeeping associations
Stephen	Hobbyist	38	Male	1	Some college, no degree	$100-150K	Moderate	Suburb	Active, attends meetings
Tommy	Hobbyist	22	Male	4	Bachelor’s degree	$75-100K	Liberal	Urban	Minimally active, reads Facebook page

^1^ Years beekeeping.

^2^ Elliot chose to submit a written set of responses in lieu of a full interview.

^3^ Not available.

Five interviewees reported having beekeeping-related careers including employment at Best Bees, nonprofit organizations, and other nongovernmental and governmental institutions that do not fit neatly into common beekeeper categorizations. Nearly all beekeeper interviewees, with the exception of Marigold and Blake who trained at Best Bees, attended *bee school* led by a county-level association.

Demographically, ethnographic observation suggests that much of the socially active eastern Massachusetts beekeeping community is racially white; no nonwhite beekeepers were noticeably observed at events and all interviewed beekeepers identified as white, except one individual who identified as Middle Eastern (Table 1). Further, most beekeepers at association events appeared near 50-years-old—the approximate interviewee median age. Best Bees employees, however, reported that their company is largely under age 30, leading some employees to perform boundary work with the wider association-centered community due to these age differences. Many employees at Best Bees also identify as LGBTQ and some explained that they feel association meetings are largely attended by heterosexual couples. These two beekeeping communities do, however, interact. For example, the Boston Area Beekeepers’ Association once gave a research grant to Noah Wilson-Rich (the founder and owner of Best Bees), while interviewee Brooke, a 54-year-old white female hobbyist, is both an active member of her local association and a client of Best Bees. Multiple beekeepers not associated with Best Bees also indicated that they have seen one or both of Wilson-Rich’s two popular urban beekeeping-focused TED Talk videos (circa 2012 and 2019) [151, 152].

## Results and analysis

### Perceptions of environmentalism: Environmental awareness and pollination providers

*We really buy into the fact [beekeeping is] a great thing to do for the environment*. *The bonus of course is the honey*, *but we really got into it for the environment* (Brooke).

Hobbyist beekeeping in Massachusetts is deeply entangled with intentions for environmental conservation. The Best Bees website claims that by using their services, clients have a *positive* and *tangible* impact on the environment. Four interviewees and three bee school students reported “helping the environment” as a major motivator for their beekeeping practice (Table 2).

**Table 2:** Beekeeping as helping the environment.

Source	Representative Quotes
Kitty	*I think it [beekeeping] should be more widespread because the primary reason is what’s happening to our environment*.
Helene	*[Beekeeping is] not our livelihood*, *we’re trying to do it for the environment…we would be a classic hobbyist*.
	*[The most rewarding part of beekeeping is] doing good things for the environment*.
Marigold	*…we’re [The Best Bees Company is] doing this great thing for the environment…*
Bill	*I like that I’m helping put bees back in the environment*.
Bee School Student	*Help myself and help nature*.
Bee School Student	*I am a science teacher*, *after teaching my kids about the bee decline and the importance of bees in our ecology…*
Bee School Student	*She’s in it for the bees*, *the honey*, *helping the environment…*

These data illustrate how common the beekeeping as environmentalism narrative is in this community. These responses further show how contact zones among social organizations are powerful sites of narrative reproduction regarding honey bee pollination and conservation or environmentalist goals. For example, Harvey, a 60-year-old sideliner, explained that a local nonprofit organization invited him to relocate a few hives to their conservation land because they believed this aligned with their organizational *mission*. A similar situation is illustrated in the Winter 2018 issue of *The Massachusetts Bee*:

“The Bee City program is operated by the Xerces society as a way of promoting and certifying towns and campuses that actively promote and encourage pollinators. Although it refers to bees…they intend to encourage all types of pollinators—monarch butterflies and the lot…I have been talking with friends on the Lexington Conservation Commission…about actively promoting pollinators. Lexington…is very receptive to this idea and has encouraged me to place honeybees on town properties. They even intend to purchase some additional honeybees for me this spring for our community garden…”

This excerpt thus illuminates how relations with social institutions can powerfully reinforce the predominant narrative of beekeeping as an environmentalist action, supporting the Capitalocene approach to resolving environmental problems by solving pollination with commodified honey bees. These contested spaces are complex and power-laden; for example, even after reference to the Xerces Society’s focus beyond the honey bee, beekeeping is still offered as the proposed solution to this crisis. This excerpt also demonstrates that the broad narrative in which beekeeping is seen as beneficial for the environment can then be grouped into two highly entangled sub-narratives: (1) beekeeping fosters environmental awareness and (2) honey bees help the local environment by providing pollination services in the Capitalocene.

#### Catalysts for environmental awareness

Interviewees and *bee school* students provided three explanations for their belief that beekeeping increases environmental awareness. Beekeeping fosters a greater awareness of: (1) general environmental conditions; (2) problematic environmental conditions; and (3) native bees specifically, sometimes even leading to conservation actions (Table 3).

**Table 3:** Narratives that beekeeping fosters environmental awareness.

Environmental awareness sub-narrative	Source	Representative Quote
(1) Fosters greater awareness of general environmental conditions	Sofia	.* *.* *.*helping the environment because you’re helping to keep the honey bees alive*. *But*, *even more importantly*.* *.* *.*you have to learn about temperature*.* *.* *.*weather*.* *.* *.*what is growing in your area…worry about daylight and wind and rain…You’re just more conscious about what the Earth is doing*. *It’s not just the bees*, *the bees are sort of a conduit of catalyst for learning the other stuff too…*
	Ambrose	.* *.* *.*If you become a beekeeper*.* *.* *.*you start to look at plants to see what bees are working and what’s flowering*, *so you don’t miss those flows…I will look at plants more than the usual person probably does*. *I’ll look and I see that bumble bees and bees are working the same flower a lot*.* *.* *.
	Elliot	*…beekeeping is overall good for the environment*.* *.* *.*by getting people who are interested in bees and those close to them to develop a closer connection to the environment*.
		*Indirectly*, *bees are helping the environment by making beekeepers…more connected to the environment*, *which makes them care more about it…I…pay more attention to my actions since becoming a beekeeper…family and friends have come to respect bees’ roles as well*.* *.* *.*With more beekeeping I would expect*.* *.* *.*more environmental consciousness*.* *.* *.*less people misunderstanding insects*.
	Bee school instructor	*You’re going to become more in tune with the environment*.
	*The Pollinators*	*They’re [honey bees are] the canary in the mineshaft*.
(2) Fosters greater awareness of problematic environmental conditions	Helene	*The environment is changing everything…and bees are the canary in the coalmine*, *I really believe that*.
	Jasper	*Honey bees just happen to be the canary in the coalmine because we use them commercially and stuff like that*.
	Kitty	*If I lose 45% of my bees*, *it makes you more in-tune with what is happening in our world*.
	Sabrina	*I’m learning that*.* *.* *.* *. *the more conscious people are of the honey bee*, *it’s actually helping*.* *.* *.*other bees*. *You know*, *the smaller bees*, *the ground bees…*
(3) Fosters greater awareness of native bees, sometimes leading to conservation action	Bill	.* *.* *.*most hobbyist beekeepers*, *if they’ve taken a class… they’ve also gotten information about helping the native bees…things you can do in your yard…I tell people…don’t plant grass*, *plant clover or other things*. *Don’t clean up all the leaves in the fall*, *leave leaves out there because a lot of the native bees nest in the leaves*. *Don’t pull dandelions in the spring because it’s one of the first food sources*.
	Kitty	*It [beekeeping] helped me be aware of what variety of bees that are…they all have a function…You need a diverse type of insect*, *including the bumble bee*, *and the wasp*, *and the yellow jacket*.

In addition to fostering greater environmental awareness in general, interviewees also explained that beekeeping leads to an increased awareness for problematic socio-environmental conditions. This sub-narrative is further illustrated through specific discussions of suburban development and landscaping practices. As Julie explained, for example, it greatly disturbs her when developers *cut down everything and put in just lawn*. *So*, *there’s not anything [left] for the bees…*(Table 4).

**Table 4:** Suburban land development and landscaping practices in Massachusetts through the lens of bee and insect needs.

Lens	Source	Representative Quotes
Land development practices	Helene	*We moved to this area because there was land…on the other side of town*.* *.* *.*one day*.* *.* *.*every tree on two-eighths of land was cut down*. *The town said nothing about it*. *Absolutely zero*. *So*, *I blame the town*.
		*It’s not just tax income*. *It’s the world*. *It’s the earth*.* *.* *.*that bothers me as a beekeeper*.
		*They don’t care if five-trillion insects were dug up*.* *.* *.*how many insects could have been killed*?
	Archie	*There are only a few of those [fields] left*, *they’re going to become houses soon…land is for sale*.
		*I just grieve for all the insects I used to remember as a kid…walking sticks and leaf walkers*, *things that you see so few of now*.
	Hyde	*These people who are like ‘oh we want to build all the housing here and destroy all the good forage’…it’s selfish to not think about other things except for money*. *It’s greed…*
	Nick	*There was so much woods*. *It was amazing…now half of that stuff is gone*.* *.* *.*[Robert] Kraft bought the land*, *bought the houses*, *leveled it*, *and it’s parking for the [New England Patriots’ Gillette] stadium now…You’re seeing that in a lot of places …contractors and people buying homes…Level the property*. *Take everything down*. *You got an acre*? *Clear it…then come in and plant trees*. *That does not register for me…*
		*The town was nice and country*, *it’s no longer that way anymore*. *It’s really changed a lot*.* *.* *.*since we moved here in ‘54…Is it good*? *Some people will say yeah*, *but I won’t*.
Landscaping practices	Stephen	*[After] being aware of…not just honey bees*, *but other bees like mason bees*, *what they eat and what they go for*, *I was like*, *why*?.* *.* *.*Why have we made our world completely sterile*? *Even down to the flowers that some people use*. *Hydrangeas—they look great but they’re sterile; there’s nothing to them…I drive around…the whole neighborhood is sterile*.
	Jasper	*Green grass is like a desert [for bees]*.
	Liam	*A lot of the flowers that people grow*, *they think oh I have a flower bed and the bees are going to [come]—no they won’t*, *because those flowers are bred for appearance*, *many of them are infertile*, *there’s nothing for the bee*
		*If there is a problem for pollinators*, *its lack of forage…You gotta have weeds…But people go to great lengths to eliminate weeds from their lawn… And like I said*, *bumble bees live in holes in the ground*, *and people dig up the ground and plant lawns*.
	Jackie	*I wish my husband would just let the clover go because there is clover and the bees love it*, *but he mows it…he wants his lawn*.
	Isabella	*He’s [her husband] kind of a fanatic about the lawn*, *which is concerning to me because there isn’t a dandelion to be found in my yard*. *No clover*. *No dandelions*. *It looks like the greenest golf course*.

These responses highlight how a beekeeper’s perspective can lead to awareness of the importance of wooded landscape conservation and the challenging of other common suburban landscaping practices, like planting monoculture lawns, for meeting the needs of less visible organisms. As beekeepers engage with local land norms through the lens of honey bee and other insect needs, even when oftentimes privileging Capitalocentric narratives, they thus illustrate how the pollinator crisis is reproduced through highly contested, local and socio-environmental processes.

Related to these claims, some also emphasize that participating in beekeeping specifically increases native bee awareness—a claim that is also supported in these data. Email communications and participant observation with MassBee confirm that native bees are a common topic—six out of ten issues of *The Massachusetts Bee* mention “wild bee[s],” “native bee[s],” or “native pollinators.” Stephen, a 38-year-old hobbyist, explained that his awareness of native bees increased due to beekeepers’ association Facebook posts. Leo, a 74-year-old sideliner, similarly explained that many speakers at association meetings discuss native bees and native pollinators. While these data cannot confirm a direct causal relationship, therefore, they do support the suggestion that beekeepers may be more likely to have an increased awareness of the needs of local native bees.

In addition to increased environmental and native bee awareness alone, multiple interviewees also explained material actions they have taken for native bee conservation. Archie, for example, leaves strips of un-mowed lawn, which he calls *bee highways*, to allow clover and other lawn flowers to grow. Bill, a 71-year-old hobbyist and beekeeper at a nonprofit organization, further explained:

*I have grass, but I don’t really take care of it. I push people to do away with grass and go with clover…The bees love clover and it does much better than a lawn…you don’t have to do anywhere near the maintenance that people do on lawns with chemicals and stuff*.

Jasper, a 60-year-old sideliner, also elaborated on a similar approach:

*I have the worst lawn in my neighborhood. I plant…a wild flower garden…not for my bees…there’s 400 types of bees in New England…multiple species of bumble bees… squash bees, green bees, there’s bees like crazy…*

Here, both Bill and Jasper first frame their approaches to landscaping as undesirable—as not *take[ing] care* of the lawn and having *the worst lawn*. In these responses, therefore, Bill and Jasper feel that to encourage their landscaping practices, they must first allude to the previously discussed social norm in Massachusetts that homeowners maintain green monoculture grass lawns. They then demonstrate how some beekeepers are actually applying their knowledge toward conservation-driven actions—even those which they see as challenging local norms.

In addition to individual actions surrounding landscaping practices, some beekeepers even come together and attend the annual Massachusetts “Ag Day on the Hill” to support state bills that place restrictions on pesticide use. For example, Brooke explained that she attended with MassBee last year because there is a *pollinator crisis*, and *something needs to be done*. The Winter 2019 newsletter of *The Massachusetts Bee* further lists sixteen related bills to support, including iterations of “An Act to Protect Massachusetts Pollinators.”

### Local pollination providers

Narratives which frame beekeeping as environmentalism are also deeply interconnected with narratives surrounding honey bees and pollination. Demonstrating the prevalence of pollination in MassBee discourse, the tenth most frequent word in our sample of *The Massachusetts Bee* is forms of the word “pollination” (S2 Table). Ten bee school students and five interviewees even reported that their main motivation to become beekeepers was largely to ensure the pollination of personal gardens and orchards—one component of a diverse and resilient New England food system that must not be overlooked [153] (Table 5).

**Table 5:** Beekeepers reporting pollination as major motivator in apiculture practice.

Source	Representative Quotes
Jackie	*I’ve always been interested in pollination*.* *.* *.*I have a huge raspberry patch*. *We initially got bees strictly for the pollination*. *We didn’t have honey for the first five years*, *and I didn’t care*.
Bill	*I started with*.* *.* *.*a mason bee*, *which is a solitary bee*, *because I wanted to make sure the garden got pollinated*.* *.* *.*I was reading a book*, *and it had honey bees*. *I said*, *‘Oh if I’m going to do bees*, *why not get honey*?*’*
	*We hadn’t put a garden at our house for years and I finally put one in*, *and I wanted to make sure it got pollinated*. *So*, *we started with mason bees and then*.* *.* *.*went into honey bees*.
Emmett	.* *.* *.*we live…my house*, *my brother’s house*, *my dad’s house*, *and there’s about 20-some apple trees*, *pears*, *blueberries*, *grapes*. *There’s all sorts of fruit that we figured would benefit from pollinators right in the backyard…*
Nick	*When I was dating my wife*, *[her] uncle at one of the [nearby] homesteads…had bees*. *We had gardens when we first married…over by their property and it was awesome*. *When they passed away*, *the hives disappeared—no one took them over…We’ve had gardens ever since*, *but it’s been going down*, *down*, *down*, *and down*.* *.* *.*I ended up getting bees and it’s been kind of leveling off now as far as produce*.
Beekeeping students:	
(a)	*Pollinization [sic] and everything like that*.
(b)	*We do have a vegetable plot and that would apply something for them to play with in their spare time…*
(c)	*We have lots of gardens*.
(d)	*I have a lot of flowering plants and shrubs*
	*I’ve noticed that in the past few years the bees in my area have dropped off*, *especially the fruits and vegetable plants*, *especially last year my yield was much lower than in the past*.
(e)	*We do some gardening*, *have some fruit trees*.* *.* *.
(f)	*We do a lot of gardening; we get the pollination*.* *.* *.* *.
(g)	*We’ve been dabbling in vegetables*.
(h)	*I now have a field so I can have bees and I grow berries and vegetables*.
(i)	*I have a substantial garden and a good wetland area*, *since we’re both into herbology*, *we hope to get more than the honey*.

Regardless of how these respondents came to accept the concept of the pollinator crisis as valid and applicable to their surrounding landscapes, these responses clearly illustrate that beekeepers see apiculture as a way to adapt to a landscape that is unable to adequately provide pollination. Increasing pollination is then led to be framed as a form of environmentalism; Helene, a 71-year-old hobbyist, explained: *beekeeping helps the environment in an inadvertent way*…*when bees pollinate*. Emmett, a 36-year-old hobbyist, further demonstrated this narrative:

*Honey bees are a breed of insect that are…specifically designed to pollinate. And they’re really, really good at it…In that regard…every plant that those things touch is…helped…*

*As far as motivation… we’re pretty evenly split between…helping the environment versus honey is really awesome… that’s basically it, you know? The pollination is important, and honey is awesome*.

Here, Emmett draws a direct parallel between helping the environment and the pollination of fruit trees on his family’s property. Isabella, a 42-year-old sideliner, similarly explained her perception of *contributing* to the environment through honey bee pollination as a major reason she continues to beekeep:

*You need to [beekeep] for other reasons…I feel like I’m contributing…to the environment by having the bees…neighbors…[have] said, ‘Oh my gosh…I’ve had the most apples I’ve ever had on my apple tree.’ You know? People are actually grateful that I have the bees, which is nice*.

As Isabel recounts not only her own perceptions of honey bees benefiting the landscape, but the importance of her neighbors’ agreement in this regard as well, she illustrates how the perception of beekeeping as environmentalism is continually reproduced through relational and power-laden socio-environmental processes.

These data thus illustrate a powerful belief among beekeepers that increasing honey bee pollination in the landscape is a “win, win, win” (*The Pollinators*). Marigold and Blake, both Best Bees employees in their mid-20s, explain respectively: *As far as pollination goes*, *the more bees the better*, and *I think the more pollinators we get out there*, *the better*. When instrumental logic is applied to describe the problem of the pollinator crisis, therefore, the crisis can then be solved, and the *environment* thus *helped*, by simply increasing pollination through introducing honey bees.

Many of these responses, therefore, rely on a mode of thought which frames pollination as a generalizable ecosystem service that exists independent of any specificity or hybrid ontology, as Brooke explained:

*To me, I don’t think it matters. I’ve had discussions with people and even the honey bee is not native to our society…we definitely have a whole bunch of species of pollinators and bees in Massachusetts, but as far as specifying native over—I just think pollinators in general need protection*.

This rationality thus acts to obscure the many relational specificities that wild native bees and the wider landscape emerge from.

Increasing honey bee populations as a form of environmentalism, therefore, is a response to the pollinator crisis that can be characterized by the instrumental logics of Capitalocene environmentalism. Given that these logics are the same as those foundational to how capitalism encourages engagement with the landscape, actions like increasing honey bee populations to improve the pollinator crisis are also likely to result in the unintended consequences characteristic of the “second contradiction” [52]. These discussions, however, are further complexified by a high awareness among some interviewees regarding the importance of native pollination in the landscape (Table 6).

**Table 6:** Importance of native bee pollination.

Source	Representative Quotes
Elliot	*Bees all pollinate different kinds of plants*, *so a wider variety of bees means more active and successful pollination*, *as well as richer biodiversity*, *and more links in the food chain*.
Helene	*I know there are hundreds of kinds of bees…native bees*, *which people don’t know about*. *They have no idea there are native bee pollinators*. *I belong to the wildflower society*, *so you see native pollinators sometimes in their pictures on indigenous plants…I’m looking for it*, *but I think the average person doesn’t have a clue*.
Marigold	*…honey bees aren’t the only pollinators*, *in fact they’re some of the fewer pollinators…out there…not the bulk of the pollinators*. *Native bees and butterflies and birds and all of that…are also going through the same issues*.
Jasper	*What’s disturbing…about the native bees…nobody even knows that the species exists and then they’re dead…There’s probably some plants that don’t get pollinated anymore in New England because that very specific fly or bee has been wiped out…But at least they’re trying today to get a…good picture of what we have*. *There’s some changes*. *I finally saw a lady slipper for the first time in years…we killed the pollinator—there was a very unique pollinator*, *we don’t even know what one it is*, *and all of a sudden there weren’t any lady slippers anymore*.
Kitty	*…variety of bees…out there and they all have a function*.* *.* *.*I have an Italian plum tree and we just can’t wait for the thing to flower…but if the little*, *teeny tiny fly that pollinates it doesn’t show up*, *we have no plums because no other insect will pollinate it*. *You need a diverse type of insect*, *including the bumble bee… wasp… yellow jacket*.
Stephen	*…There’s so many different ones…little mason bee that no one really knew about…looked like a fly*, *ended up being the only thing that pollinated your—I don’t know—your lilies*. *And then next year*, *you’re wondering*, *‘why didn’t my lilies come back*?
Bill	*The other thing we’re trying to do is encourage native bee*, *expansion*, *so people doing things to help the native bees*. *Native bees are much better pollinators than honey bees*.

These comments show that while an instrumental framing of pollination was most prevalent in beekeeping discourse, some interviewees also illustrate deeper engagements with the implications of relational and contested landscapes where specific contact zones are essential for supporting biodiversity. As we discuss in the next section, these viewpoints will only become more important to encourage in order to avoid and mitigate the unintended consequences that become more likely as environmentalist approaches are applied in the Capitalocene.

### Unintended consequences and socioecological interpretations of invasiveness

*The honey bee is an invasive species in the United States*…*That’s why the Xerces Society doesn’t like honey bees*…*Xerces tries to bring species*…*natural to…America…Not the invasive plants*…*invasive insects*…*invasive animals*. *I understand where they’re coming from*…*We need those kinds of people*, *right*? *They used to promote honey bees and then all of a sudden…five or six years ago they went ‘honey bees are evil* (Jasper).

When asked if he believes beekeeping should be more widespread, Emmett responded: *I actually have mixed feelings on that…they are technically an invasive species*, *right*? *The strains we’re using are Europeans*…*my bees are Italians*. When queried further about previous encounters with the word *invasive* and honey bees specifically, Emmett then replied:

*Non-native, I guess I’m supplying invasive. But isn’t that how invasive species happen?…It’s a good idea to bring this over here and now all of sudden they’re here for good or ill…Normally invasive has a more negative connotation, no one at bee school has a negative view of bees…more in terms of, gosh how the heck do we keep honey bees alive in a New England climate…our growing season…forage season, is pretty short, and the winters are long and harsh, and these things kind of like a Mediterranean environment because they’re Italian*.

This response demonstrates an awareness of the problems that often lead nonnative species to be considered invasive—though Emmett remains seemingly unaware of the specific reasons why this connotation might become associated with honey bees. In a similar way to how Jasper denaturalizes the term *invasive* in the quote at the beginning of this section, though, Emmett’s commentary on the often-unintentional nature of species invasions represents a powerful grappling with *invasive* as a contested label. Jasper once again highlights how conservation organizations, in this case the Xerces Society, are actively entangled in contested socio-environmental processes through which species come to be considered invasive. We will first discuss how this process is illuminated through discussions of honey bee invasiveness in the context of interspecific bee competition.

#### Concern and skepticism for the effects of interspecies competition

Concern for sufficient forage to support bees is a common topic among beekeepers. Blake explained: *One of the biggest issues…bees and native pollinators are having right now is land… losing resources*. While some competition among honey bee colonies (intraspecific competition) was more common in discourse overall (S3 Table), some interviewees also expressed an awareness of concerns for interspecific bee competition (Table 7).

**Table 7:** Concern for interspecific bee competition: Skeptical to concerned.

Source	Concern Level	Representative Quotes
Elliot	Skeptical	*…an argument highlighting how beekeeping adds competition to native species of bees*. *I mostly disagree…I believe that overall beekeeping is good for the environment*, *by bolstering pollination (not outcompeting natives)*, *and by getting people…to develop a closer connection to the environment*.
Archie	Skeptical	.* *.* *.*a study done in England…keeping so many honey bees*, *they were stealing all the pollen and nectar resources from the wild bees*. *I’m skeptical*. *I’m skeptical because there are a lot of flowers out there…different bees want different flowers anyway because bees in the Western hemisphere are imported*.* *.* *.*They’re an invasive species*, *so they…often prefer plants… also…from Europe*.* *.* *.*A lot of weeds that came over in bags of seed and trees*.* *.* *.*are not native here*.* *.* *.*I notice that in our yard*, *we see wild bees go to one plant and honey bees go to another plant*, *so I’m skeptical*.
Blake	Skeptical	*Some people have expressed concern about honey bees out-competing or harming native pollinators…We haven’t found much evidence that that is a real issue…Maybe*.* *.* *.*a bumble bee and a honey bee meet on the same flower*, *there might be a little competition*.* *.* *.*through that tussle*, *there can also be a transference of pollens…simple bumping can spread even more pollination…I think potentially there could be an issue with competing*, *but if there’s enough resources to go around*, *it’s not an issue*.* *.* *.*I don’t know*, *I don’t think there’s enough research on it to know for sure*.
Harvey	Skeptical	*I see lots of other bees*, *all seem to be getting along fine…two or three different bees on a single plant*, *they’re getting along fine*. *They’re not fighting*. *So*, *I don’t observe a problem*, *but I don’t know*.* *.* *.
		*I think they go for different plants; bumble bees don’t go for the same plants as honey bees*.* *.* *.*they go for different things at different times*.
Marigold	Mixed	*I’ve heard some argument that domesticated honey bees…too many of them…would be taking over*. *It’s kind of an invasive species for the native bee*, *preventing them from having the resources to thrive*.* *.* *.*Some article in Science*.* *.* *.*said…[beekeeping] was detrimental to native bees*. *I don’t know*. *I would have to do more research to figure that out*.* *.* *.*It’s definitely something that people talk about…*.*wonder about*. *But is there enough research to back it*? *I have no idea*.
		*I do think that too much of a good thing can be not a good thing…backyard beekeeping should increase*.* *.* *.*and it is*.* *.* *.*So ideally everyone have some bees—well not everyone…But enough people want bees that will warrant stricter registration and record keeping on the number of bees in an area…I do think you can ‘over-bee’ an area*, *but I don’t see that ever reaching a point…not in the near future…*
Stephen	Mixed	*My neighbor’s tree…these big puffy white flowers and everything’s on there*.* *.* *.*Big black wasps*, *giant wasps*, *to mason bees*, *to honey bees*, *to bumble bees…They’re almost crawling all over each other…They don’t care when there’s food…I’m getting food*, *you can get food…who cares*? *But I think when the flowers stop that’s when you’ve got to start looking…*
Tommy	Concerned	*I try to be an advocate for urban beekeeping*. *But*.* *.* *. *there are native bees that are competing for resources as well*.* *.* *.*honey bees are an invasive species*, *we brought them over from Europe…all that kind of sits in the back of my mind*.
		*If everyone kept honey bees on their back porch the same way as a I do*, *there would be a huge dearth of resources and that would be bad*.* *.* *. *I should…introduce some more resources…small planters with diverse*, *native flowers on my back porch…Introducing insects that are going to consume resources and leave less resources for native species…*
Hyde	Concerned	*…Why do we need 40*,*000 foragers*? *Most of them are…in the colony doing nothing…They interact then with the Bombus…and other orchard bees…they don’t get as many nutrients…They found out that the size of these bumble bees are actually smaller than they would be if the Apis mellifera colony was taken away*. *That’s a big deal*.* *.* *.*It’s scary*.

Among those aware of interspecific competition concerns, most expressed ambivalence and skepticism that this type of competition is a real threat to wild bees. First, possibly due to receiving similar messaging from Best Bees, skepticism expressed by Blake and Marigold emerged from the belief that there is not enough scientific evidence to consider interspecific competition a threat. Due to the host of entomological literature that would likely disagree with this interpretation, this response thus illustrates a possible example of the how “probabilistic science” may obscure the socio-environmental processes through which biodiversity becomes suppressed [130].

Remaining skepticism in these data can be further connected to Capitalocene logics. To illustrate, Elliot, a 20-year-old hobbyist, explained that honey bees *help the environment by bolstering pollination*, *not outcompeting natives*. Seeing pollination as an independent and easily substitutable ecosystem service, Elliot is thus able to cognitively compartmentalize pollination as a separate process from the gathering of pollen and nectar for consumption. This logic thus effectively obscures the fact that pollination is not truly a separate process, but deeply entangled with other processes that sustain a deeply complex web of life.

Skepticisms illustrated by Harvey and Archie are similarly rooted in a predictable, instrumental framing of the landscape. The logic that honey and native bees do not rely on the same plants, and thus, concern for interspecific competition is eliminated, frames honey bees and plants as predictable and stagnant species. Plants are especially seen as passive recipients of pollination services. This logic thus ignores and obscures the distributed agency and power that bees, plants, and all “things” have as they become-with each other and shape the landscapes with which they emerge. The plant community, for example, is deeply entangled in power-laden process of growing and changing, or “becoming-with,” the local multispecies community—a topic that will be further explored in a later section [6, 51].

Finally, Tommy, a 20-year-old hobbyist, and Hyde, a 25-year-old hobbyist and employed beekeeper, represent those who were greatly concerned that competition may have harmful impacts on native bees. For example, after hearing about competition concerns while attending a presentation at the Harvard Arboretum, Tommy began to consider introducing containers of native flowers to support native bees. Hyde also explained that some beekeeping practices, like keeping large colonies and having many apiaries in one area, are additional factors that must be considered:

*In nature…[bee colonies] are usually a quarter mile away from each other…[and therefore are] not quite interacting with…Bombus or other honey bees, solitary bees, orchard bees—they still have a region…,they can get around…These colonies…are not the size of colonies that we have. They’re smaller…So, there’s less bees as well*.

As Hyde suggests, therefore, there may be ways to change beekeeping norms—including keeping smaller hives and being more attentive to the number of colonies in an area—to better account for the unintended consequence of interspecific bee competition. The former suggestion, as Hyde elaborates, would likely require that Langstroth hives be replaced with smaller, alternative hive configurations—thus illustrating how the material hives that bee colonies live within are also important agentic things in the landscape. The latter, as we discuss further in the conclusion, however, would require a highly collaborative social context open to beekeeper registration and eventually regulation—topics of great controversy in the beekeeping community.

#### Pathogen transmission and the hybridity of health

In Massachusetts beekeeping discourse today, concern for honey bees is overall dominated by *Varroa destructor*. *Varroa* is seen as “public enemy number one” (Dr. Samuel Ramsey in *The Pollinators*) and “the enemy of the honey bee and the biggest problem beekeepers face today” (Norfolk County Beekeepers’ Association brochure). Beekeepers are highly aware that *Varroa* can spread via interactions among colonies and local apiaries; colonies with high populations of mites can lead to the collapse of nearby apiaries, referred to as *Varroa bombs*. Most members in the community treat for *Varroa* with a chemical miticide, often in the form of oxalic acid with brands like Formic Pro® or HopGuard®II, though there are also advocates for using less hazardous substances like rhubarb leaves and mushroom extracts. These contested exchanges and dynamics that surround competing views on *Varroa* treatment are illustrative of the local socio-environmental coproduction of *Varroa* as a major concern.

*Varroa* is not only a threat to honey bee health because it feeds on honey bee fat body, which is directly responsible for immune system functioning, but also because it acts as a vector for pathogenic organisms to travel across bee bodies [154]. As one speaker at the 2019 MassBee meeting explained: *viruses are driving the Varroa bus*. Beekeepers and honey bees are thus riding a *Varroa bus* driven by obligate pathogens—a strikingly hybrid scene where agency is clearly distributed across a muddled landscape of things.

In our engagements with beekeepers, we find that this emphasis on *Varroa* for honey bee health is also foundational to beekeeper discourse on health concerns regarding native bees. To illustrate, when asked what he has heard about interactions between honey bees and wild bees, Liam, a 77-year-old sideliner, said:

*There’s some very thin science about that. A lot of opinions, no replicated studies…Obviously, if the bees were on the flowers, they’re going to pass their bugs around, pass their mites around. I mean that’s how my bees come home with mites…*

Here, *Varroa* is thus discussed as an independent actor in the landscape. In a similar way that instrumental logic is used to understand honey bees in the landscape, then, this same logic is applied toward understanding *Varroa*. This logic thus obscures the reality that *Varroa* exists embodied in a hybrid landscape of not only other insects, but also both prokaryotic and eukaryotic microscopic organisms that surround and live within *Varroa* and bee bodies. Ambrose, a forty-one-year-old sideliner beekeeper, is similarly unable to take into account of these hybrid contact zones. In contrast to Liam’s confidence, however, Ambrose demonstrated a real grappling with the possibility that there are negative interactions of which he was unaware:

*We know that mites hop bees. But I’ve always wondered if those mites are going after the bumble. If not, why aren’t they doing that?…I mean we know that the mites are going after the body fat…underneath the plates…maybe the bumble bees don’t have that same kind of body…*

This response thus illustrates an inclination to focus on contact zones and relations, but only at a species level that maintains a view of individual actors in the landscape. This then obscures the reality that managed hives may build up microorganism populations that can then spread to wild bees via other vectors, like the surfaces of flowers. Only two data sources went beyond this mode of thought to discuss bee and landscape health with a hybrid lens.

In the Winter 2019 issue of *The Massachusetts Bee*, an article titled, “Flowers as “viral hotspots’” draws attention to recent findings that honey bees can transmit viruses onto the surfaces of flowers [155]. The newsletter article concluded that this research “is an important step in understanding disease transmission between bee species.” Hyde also discussed pathogen transmission via flower surfaces, but within the context of a more direct discussion of interspecific transmission from honey to bumble bees:

*Viruses that honey bees have…have both a horizontal and a vertical vector…[Honey bees] can give each other the viruses…that’s the vertical…The horizontal is, if a honey bee goes onto a flower and a bumble bee comes onto it, the bumble bee can then contract the viruses…We know if there are honey bee colonies in an area, that bumble bees in that area are also affected by the viruses…It’s a big issue…This is going from Apis mellifera to Bombus…This is a big deal and we’re not talking about it*.

Hyde not only expresses an awareness of interspecies transmission here, but also concern that this topic is rarely discussed in the beekeeping community. This reveals a need to increase awareness surrounding these hybrid issues that involve powerful nonhuman actors [78], while at the same time, also illustrates that it may be difficult to present this information in a way that would be socially accepted or acted upon by the beekeeping community.

Beyond discussion of pathogenic microorganisms alone, Hyde goes on to connect honey bee health to a wider view of landscape health as founded in the microscopic communities that become-with healthy soil:

*Bacteria, protozoa…mycelium…living organisms…It’s a world right there…If I have a nutrient-filled soil…the soil will be able to give more minerals and nutrients to the honey bees…A healthier bee…This is how I think about my beekeeping. It’s not just about the colony. I’ve spent the last six years studying the colony…studying the behaviors…‘What management techniques are the best for what I need to do?’ And that’s cool. But one of the reasons that I took a step back…was to look at the health of the soil…What can we do on the outside [of the hive]?*

This discussion exemplifies an even deeper understanding of the hybridity of health in relational landscapes—one which also offers a suggestion for creating more just landscapes for the future. Not alone in this sentiment, agroecologist Dr. Lundgren says in *The Pollinators*: “A healthy soil…This is the answer to the bee problem. If we got this on most of American’s soils again, your bees would stop dying” [156]. These data thus illustrate how hybrid perspectives do not result in instrumental solutions, but in solutions that seek to cultivate the socioecological conditions most conducive to biodiverse landscapes as a whole.

#### The socioecological dynamics of plant communities

The problematic consequences of instrumental logic as it moves across contested socioecological landscapes is further illustrated through discussions of beekeeping and local plant communities. At the MassBee Spring 2019 Meeting, a speaker representing MDAR explained that the organization will soon be able to analyze the pollen gathered by honey bees to learn from which species they most often forage. The speaker then said: *I expect a lot [of plant species] will be invasives*. Archie, Liam, Bill, and Jasper also discuss that honey bees often forage on introduced plants that are widely considered invasive in Massachusetts. They specifically mention two species, purple loosestrife (*Lythrum salicaria;* Lythraceae) and Japanese knotweed (*Reynoutria japonica;* Polygonaceae). Both plants are widely recognized as invasive throughout North America—including by the Massachusetts government, UMASS extension, and the Audubon Society [157–159]. Both plants are also considered especially problematic in disturbed areas surrounding wetlands; the MA Department of Conservation and Recreation describes purple loosestrife as “a hardy, aggressive, non-native wetland invader” [160]. An MDAR representative at the Pollinators in Our Land Conference similarly explained that Japanese knotweed has created *ponds you can’t even get into*.

Japanese knotweed reproduces largely through vegetative asexual reproduction—making honey bee pollination immaterial for immediate spread, though still important for genetically diverse and stable plant populations in the long term [161–163]. Lacking in this ability for asexual reproduction, however, the relations between *Apis mellifera* and purple loosestrife may be more imminently concerning; *Apis mellifera* has been found to be the main pollinator of purple loosestrife when growing in North America [63, 161, 163].

While most label these species as invasive, some beekeepers provide a different narrative. For example, Jasper and Liam both champion these species for honey production. Jasper explained: *I get a fall nectar flow where I can pull honey off my hives once every three years*…*It’s usually an invasive that the Xerces Society wouldn’t like called Japanese knotweed*. Jasper also elaborated that purple loosestrife makes a *nice dark honey* and there was once *almost a fist fight in a beekeeping meeting* when the state government released a beetle as a biological control to remove purple loosestrife from local wetland areas.

Beyond individual desires for honey harvests, Jasper and Liam further demonstrated the contested nature of invasiveness while they explained that these plants are beneficial to insects beyond honey bees alone. Regarding Japanese knotweed, Jasper said:

*That plant, which I see along the Charles River a lot…Bees love it…all bees get on that thing. When that thing is blooming real well…not just…my honey bees. I’m talking everything. All bee species, flies, moths. There’s something good to say about it*.

Liam also discussed purple loosestrife in a similar frame:

*The neo-ecologists are pouncing on the purple loosestrife and saying that it’s invasive and we gotta get rid of it. No! Purple loosestrife blooms at the time of the year where there’s actually very little in the environment that’s blooming, so it’s a very important nectar and pollen source for not just honey bees, but for the natural pollinators…*

In these responses, Jasper and Liam once again illustrate how power is distributed across agentic assemblages of beekeepers and social groups—in this case the Xerces Society, Massachusetts government, local beekeepers’ associations, and the group Jasper refers to as the *neo-ecologists*. Relations within these groups are thus once again shown to be powerful spaces in the socio-environmental process in which species materially and discursively become constituted as invasive. Jasper further complexifies this contested labeling process through discussing purple loosestrife in comparison with agricultural wheat fields:

*It’s been here a hundred years! Come on kids! It’s part of the environment, leave it alone. If you want to talk about invasive plants that have disrupted the environment…nothing, in terms of acreage, is as bad as wheat…whole middle part of the country, tens of millions of acres of prairie…turned it into wheat fields…Wheat is okay and purple loosestrife is not?…Who gets to decide these things?*

By illuminating the power-laden ways through which species become invasive then, these data highlight that while invasiveness is often framed as a species characteristic, it is actually a dynamic and contested socio-environmental relation.

As Jasper and Liam frame plants as generalizable providers of nectar and pollen, not as agentic coproducers of the landscape, therefore, their assumptions and modes of thought are once again shown to be entangled into their interpretations of the landscape. This instrumental framing, however, cannot account for how insects and plants become-with one another. Blake offers an illustration of how this logic can then lead to Capitalocene environmentalism, which relies on the ability of humans to create and complete dominate the landscape:

*We definitely want to support native plants because that’s…what’s going to support out ecosystem the best. But in cities, we’ve already removed nature…we can kind of play with it a little bit and we can…reintroduce nature in the way that we want to. And I think it’s important that we do whether it’s native or not*.

This species-centric and instrumental framing thus fails to consider the hybridity of these plants in the wider landscape, and therefore, increases the likelihood for unintended consequences [Cf. 164]. For example, while these plants may offer food for multiple species in today’s novel and damaged landscapes, they are also known to contribute to the suppression of native plants, and thus can negatively impact the local wildlife community overall—especially for “specialist” species [120, 165].

## Conclusion: Toward just landscape futures

*Beekeeping is not an individualist occupation*. *The only way for the beekeeping industry to thrive in Massachusetts…[is through] cooperation between beekeepers*, *farmers*, *housing developers*, *[and] chemical companies* (Hyde).

If more just and biodiverse futures are to be realized, then beekeeping communities must escape Capitalocene logics and encourage understandings of apiculture as situated within hybrid, socioecological, and contested landscapes. With a keen awareness of these dynamics, the quote from Hyde above explains how just transitions for the pollinator crisis may include embracing a less individualistic and instrumental view of beekeeping—one in which not everyone should be a beekeeper. Hyde continued:

*People get excited by bees*, *and that’s good*…*Without that*, *there wouldn’t be this huge movement to try to create environmental conditions to help the bees…On one side*, *it’s really good to have this bee desiring culture*. *But on the other hand*, *everybody wants to do it themselves*. *There needs to be a sharing*.

This discussion recognizes the power that beekeeping networks may have in increasing awareness for bees and the wider environment. More importantly than individual actions, though, Hyde continues to explain that to truly reduce competition and disease pressures on native bees, beekeepers must collaborate to lessen regional population density. A small step toward this goal would be keeping a record of the number of beekeepers and apiaries in the state; as Hyde explained: *We need to know what’s in the state…the condition of the bees*. While multiple interviewees expressed that they are not opposed to the idea of mandatory registration, other members of the beekeeping community, however, have spoken out against this idea in recent years. When MDAR began revising its apiary regulations in 2018, for example, “mandatory registration topped the list of proposed regulations that beekeepers oppose” [166]. Nick, a 75-year-old hobbyist, explained that mandatory registration was *one of the big hang-ups* on beekeepers approving the changes:

*MassBee…we don’t want regulations…We try to get in front of it and educate, mostly the politicians, because we don’t want regulations…The problem is the dog catcher…doesn’t know anything about honey bees, so we tend to oppose any kind of legislation against bees and fees…Where’s this money going to?…No, I don’t want you to be in downtown Boston little apartment and put twenty beehives on your balcony, but I don’t want any legislation against it…Bees don’t attack you. They’re not yellow jackets, they’re not going to sting your kids. They’re not going to bother anybody, and we promote responsible beekeeping*.

As Nick illustrates here, many in the beekeeping community are against regulations because of the perception that regulations are intended to protect humans from bee stings, not local wildlife from the pressures of honey bee colonies. Reframing this topic with a central focus on the wild landscape may open more pathways for beekeepers to reconsider how registration, and regulations more generally, may be an essential component of managing concerns for honey bees in the landscape.

As Hyde alludes to above, though, any positive changes in the landscape on a regional scale would require that beekeepers act as a collaborative collective, not a community of discrete individuals. This may be difficult, however, given that one extremely common phrase in the Massachusetts beekeeping community is variations of *ask ten beekeepers a question and you’ll get twenty answers* (S4 Table). This saying first appears to be a benign claim about the plenitude of beekeeping practices. A critical hybridity framework, however, illuminates a deeply individualist undertone reflective of Capitalocene (and neoliberal) logics in which beekeepers are encouraged to be fully entitled to their own personal opinions and practices—a social condition which can effectively silence those advocating for collaborative solutions to both beekeeping-specific and conservation goals.

To achieve Hyde’s vision, therefore, approaches to creating more just and healthy landscapes must be inclusive of varied perspectives—including the wider socio-environmental landscape and social institutions like local community groups, conservation organizations, and social and natural scientists at research organizations and universities. This collaborative approach is essential if greater numbers of people are to recognize the ecological potential of their local landscapes and the importance of their own role in shaping the future. With multiple beekeepers in this study expressing care for native bees and local environmental conditions, and still increasing awareness of the importance of biodiversity for local ecosystems, this better future may be within reach.

To continue to strengthen engagement with hybrid perspectives of the landscape, we call on future research to examine the possible unintended consequences surrounding various types of biodiversity conservation and agricultural initiatives. Regarding the pollinator crisis specifically, we call for critical socioecological analysis of programs similar to the UNESCO and Guerlain’s “Women for Bees” entrepreneurship program [167] or the United Kingdom based “Bees for Development Trust” [168]—both of which claim to support biodiversity conservation and the economic empowerment for marginalized populations simultaneously. More broadly, we call for further research to consider the already-occurring ways that groups of people are responding to socioecological crises, particularly those less-visible processes like biodiversity loss among plants and soil-dwelling organisms. Especially in an era of increased precarity due to global climate change, we must be increasingly and iteratively critical of the assumptions behind our actions and the promises of Capitalocene-based logics. It is through this critical engagement with landscapes as hybrid places of mutual becoming-with that more just ways forward may be revealed.

## Supporting information

S1 Table Massachusetts beekeeping community participant observation. (DOCX)

S2 Table Word frequency in The Massachusetts Bee newsletter issues. (DOCX)

S3 Table Concern for intraspecific honey bee competition. (DOCX)

S4 Table Individualism in the beekeeping community. (DOCX)

S1 Text Interview schedule. (DOCX)
