# Supplementary material for: Be(e)coming pollinators: Beekeeping and perceptions of environmentalism in Massachusetts
Source: PLoS One. 2022 Mar 14;17(3):e0263281. doi: 10.1371/journal.pone.0263281 (PMC8920284; doi:10.1371/journal.pone.0263281)
Supplement: S1 Table — (DOCX) [file pone.0263281.s001.docx]

| **S1 Table. Massachusetts beekeeping community participant observation** | | | |
| --- | --- | --- | --- |
| **Event** | **Organization(s)** | **Date** | **Location** |
| 2018 MassBee Fall Meeting | MassBee | Fall 2018 | Bristol, MA |
| The Best Bees Company site visit | The Best Bees Company | March 2019 | Boston, MA |
| 2019 MassBee Spring Pre-Meeting Dinner | Essex County Beekeepers Association | Spring 2019 | Topsfield, MA |
| 2019 MassBee Spring Meeting | Essex County Beekeepers Association | Spring 2019 | Topsfield, MA |
| *The Pollinators* documentary | Woods Hole Film Festival | June 2019 | Woods Hole, MA |
| *The Pollinators* documentary | Newburyport Documentary Film Festival | September 2019 | Newburyport, MA |
| 2019 MassBee Fall Meeting | MassBee, Norfolk County Beekeepers Association | November 2019 | Medway, MA |
| 10th annual Langstroth Bee Fest | Town of Greenfield, 2nd Congregational Church of Greenfield, Franklin County Beekeepers’ Association | June 2019 | Greenfield, MA |
| Topsfield Fair Honey Show | Essex County Beekeepers' Association town of Topsfield, MA | October 2019 | Topsfield, MA |
| Three beekeeping classes (*bee school)* | Bristol County Beekeepers' Association | February-March 2020 | Bristol, MA |
| Pollinators in Our Landscapes Conference | UMass Extension | February 2020 | Milford, MA |
