# Supplementary material for: Be(e)coming pollinators: Beekeeping and perceptions of environmentalism in Massachusetts
Source: PLoS One. 2022 Mar 14;17(3):e0263281. doi: 10.1371/journal.pone.0263281 (PMC8920284; doi:10.1371/journal.pone.0263281)
Supplement: S2 Table — (DOCX) [file pone.0263281.s002.docx]

| **S2 Table. Word frequency in *The Massachusetts Bee* newsletter issues** | | |
| --- | --- | --- |
| Stemmed words | grouped according to word stem | (ie. “pollinators” is a representation of “pollinator,” “pollinate,” “pollination,” “pollinates,” “pollinators,” “pollination,” and “pollinated.” Size is associated with relative number of references compared to other words. |
| stop words | Non-content words (not included in the analysis) | a about above after again against all am an and any are aren’t aren't as at be because been before being below between both but by can can’t cannot can't could couldn’t couldn't did didn’t didn't do does doesn’t doesn't doing don’t don't down during each few for from further had hadn’t hadn't has hasn’t hasn't have haven’t haven't having he he’d he’ll he’s he'd he'll her here here’s here's hers herself he's him himself his how how’s how's i i’d i’ll i’m i’ve i'd if i'll i'm in into is isn’t isn't it it’s its it's itself i've let’s let's me more most mustn’t mustn't my myself no nor not of off on once only or other ought our ours ourselves out over own said same say says shall shan’t shan't she she’d she’ll she’s she'd she'll she's should shouldn’t shouldn't so some such than that that’s that's the their theirs them themselves then there there’s there's these they they’d they’ll they’re they’ve they'd they'll they're they've this those through to too under until up upon us very was wasn’t wasn't we we’d we’ll we’re we’ve we'd we'll were we're weren’t weren't we've what what’s what's when when’s when's where where’s where's which while who who’s whom who's whose why why’s why's will with won’t won't would wouldn’t wouldn't you you’d you’ll you’re you’ve you'd you'll your you're yours yourself yourselves you've page |
