# Supplementary material for: Be(e)coming pollinators: Beekeeping and perceptions of environmentalism in Massachusetts
Source: PLoS One. 2022 Mar 14;17(3):e0263281. doi: 10.1371/journal.pone.0263281 (PMC8920284; doi:10.1371/journal.pone.0263281)
Supplement: S3 Table — (DOCX) [file pone.0263281.s003.docx]

| **S3 Table. Concern for intraspecific honey bee competition** | |
| --- | --- |
| **Source** | **Representative Quotes** |
| Harvey | *You can only have so much [beekeeping] in an area. The land can only support so much.* |
| Julia | *We have so many hives now that we have some at a friend’s farm at an open clearing in the woods…We had to because our bees are like fish that multiply.* |
| Archie | *The landscaping here is kind of suburban. With the hives we have, there’s only so many flower resources for them…if we were going to have more bees, we would need a place with more wild plants than we have.* |
| Isabella | *…because I’ve had so many bees, I’m just not sure I have enough resources around…* |
