# Supplementary material for: Be(e)coming pollinators: Beekeeping and perceptions of environmentalism in Massachusetts
Source: PLoS One. 2022 Mar 14;17(3):e0263281. doi: 10.1371/journal.pone.0263281 (PMC8920284; doi:10.1371/journal.pone.0263281)
Supplement: S4 Table — (DOCX) [file pone.0263281.s004.docx]

| **S4 Table. Individualism in the beekeeping community** | |
| --- | --- |
| **Source** | **Representative Quote(s)** |
| beekeeper attendee at 2019 MassBee Fall Meeting | *People tell me fifty different ways to take care of the hive—often conflicting, but that's okay—that's the beauty of knowledge* |
| Ambrose | *Ask ten beekeepers one question, you’ll get eleven different answers.* |
| Bill | *With beekeepers you’re going to get 30 opinions.* |
| Eric | *Their mantra through the whole bee school was ask three beekeepers one question and you’ll get five answers, or seven answers. It was almost like the joke was that you never even said that the same way twice.* |
| Jackie | *…If you get ten beekeepers in a room, you get twice as many responses.* |
| Sofia | *What’s the saying? If you ask four beekeepers you get twenty answers.* |
| Stephen | *…I think that’s one of the things I’ve seen with beekeeping…asking four beekeepers, get five answers.* |
