# Supplementary material for: Be(e)coming pollinators: Beekeeping and perceptions of environmentalism in Massachusetts
Source: PLoS One. 2022 Mar 14;17(3):e0263281. doi: 10.1371/journal.pone.0263281 (PMC8920284; doi:10.1371/journal.pone.0263281)
Supplement: S1 Text — (DOCX) [file pone.0263281.s005.docx]

| **Interview schedule** |
| --- |
| **Introduction** |
| Before we get started, how about you tell me about yourself and what you like to do other than keep bees? |
| How did you decide to become a beekeeper? |
| How did you become interested in beekeeping? |
| **Beekeeping Practices** |
| How did you choose the type of hive configuration that you use? |
| Have you heard of or considered using other kinds of hives? Why or why not? |
| Where is your hive located and what is it like around there? |
| How do you care for your land? |
| How do your neighbors care for their land? |
| Did you tell your neighbors you were getting bees? How do they respond to the bees? |
| How did you decide where to get your bees? |
| Since there are debates in the beekeeping world over the use of miticides and other hive treatments, can you explain your practices and how you decided to use them? |
| What about beekeeping would you say is most rewarding for you? |
| **Beekeeping community** |
| 1. In your opinion, should beekeeping be more widespread and why? |
| 1. When you have a question about beekeeping, where do you generally go for answers? |
| - 1. How do you decide what information is reliable? |
| How did you come to be a part of a Beekeeping Association? |
| - 1. How involved are you with the association? |
| How closely does your personal approach to beekeeping align with the approaches generally taught by the association? |
| Are there any differences in you and their schools of thought? |
| Are you aware of any regulations on beekeeping in your area? |
| What is your opinion on beehive registration? |
| Do you think registration should be mandatory? |
| I’ve read that more and more young people are keeping bees but I don’t see many young people at association meetings. What have you noticed and do you agree or have any idea why this might be the case? |
| How often, if at all, do you find yourself talking about bees to non-beekeepers? |
| Where or when does that usually happen? In what context? |
| Would you say that many people you know learn about bees largely from beekeepers? |
| Are there any particular parasites or pathogens that you are concerned will be a problem, or more of a problem, in the future? |
| Have you ever heard anything regarding interactions between honey bees and other types of bees? What have you heard? |
| Have you heard the debate that keeping honey bees is not actually “good for the environment”? |
| How did you come to hear about this? |
| What is your opinion about this? |
| 1. What does the “pollinator decline” mean to you? |
| How concerned are you about the pollinator decline? |
| On a scale of 1 to 5? – 1 not concerned, 2 a bit concerned, 3 moderately concerned, 4 very concerned, 5 extremely concerned. |
| What do you believe needs to be done to address these problems? |
| Who is responsible for doing so? |
| There is a lot of talk about the importance of “native” species and “native pollinators,” to your knowledge, why could it matter if a pollinator is “native” or not? |
| Is this a common topic in the beekeeping community? |
| **Environment** |
| How much /to what degree do you agree with this statement: “Global climate change is currently occurring and is caused by human activity” ? |
| Please rate how concerned you are about climate change on a scale of 1-5? |
| 1 not concerned, 2 a bit concerned, 3 moderately concerned, 4 very concerned, 5 extremely concerned. |
| Why did you provide this rating? |
| If anything, what does climate change have to do with bees? |
| Do you find that the beekeeping community in MA that you are a part of talks about climate change? |
| **Demographics** |
| What is your age? |
| What is your self-identified gender? |
| What is your self-identified race/ethnicity? |
| What is the highest level of education you have completed? |
| How long have you lived in MA? |
| Do you read any MA or national newspapers? |
| Local newspaper? If none—how do you get your news? |
| Would you describe yourself as living in an urban center, in a suburb, or in a rural area? |
| Please select your 2018 household income and political ideology from the provided list: total household income (for year 2018): Less than $10,000; $10,000 to under $30,000; $30,000 to under $40,000; $40,000 to under $50,000; $50,000 to under $75,000; $75,000 to under $100,000; $100,000 to under $150,000; $150,000 or more; prefer not to answer. |
| 1. Political ideology: conservative, moderate conservative, moderate, moderate liberal, liberal, other answer (elaborate?), prefer not to answer. |
| 1. Do you consider yourself religious? What religion? |
| **Conclusion** |
| Is there anything that I didn’t ask that you were hoping to talk about? |
| Do you have any questions for me? |
| What’s the most important advice you would give for someone looking to start beekeeping? |
